# Smartphone-Based Rigid Endoscopy Device with Hemodynamic Response Imaging and Laser Speckle Contrast Imaging

**DOI:** 10.3390/bios13080816

**Published:** 2023-08-14

**Authors:** Youngkyu Kim, Woo June Choi, Jeongmin Oh, Kwanhee Lee, Jun Ki Kim

**Affiliations:** 1 Biomedical Engineering Research Center, Asan Medical Center, Seoul 05505, Republic of Korea; limexce@gmail.com; 2 School of Electrical and Electronics Engineering, Chung-Ang University, Seoul 06974, Republic of Korea; cecc78@cau.ac.kr; 3 Department of Biomedical Engineering, College of Medicine, University of Ulsan, Seoul 05505, Republic of Korea; mini1kr@naver.com (J.O.); gtgay@naver.com (K.L.)

**Keywords:** point-of-care, smartphone, laser speckle contrast imaging, hemodynamic response imaging, animal model of ischemia

## Abstract

Modern smartphones have been employed as key elements in point-of-care (POC) devices due to remarkable advances in their form factor, computing, and display performances. Recently, we reported a combination of the smartphone with a handheld endoscope using laser speckle contrast imaging (LSCI), suggesting potential for functional POC endoscopy. Here, we extended our work to develop a smartphone-combined multifunctional handheld endoscope using dual-wavelength LSCI. Dual-wavelength LSCI is used to monitor the changes in dynamic blood flow as well as changes in the concentration of oxygenated (HbO_2_), deoxygenated (Hbr), and total hemoglobin (HbT). The smartphone in the device performs fast acquisition and computation of the raw LSCI data to map the blood perfusion parameters. The flow imaging performance of the proposed device was tested with a tissue-like flow phantom, exhibiting a speckle flow index map representing the blood perfusion. Furthermore, the device was employed to assess the blood perfusion status from an exteriorized intestine model of rat in vivo during and after local ischemia, showing that blood flow and HbO_2_ gradually decreased in the ischemic region whereas hyperemia and excess increases in HbO_2_ were observed in the same region right after reperfusion. The results indicate that the combination of LSCI with smartphone endoscopy delivers a valuable platform for better understanding of the functional hemodynamic changes in the vasculatures of the internal organs, which may benefit POC testing for diagnosis and treatment of vascular diseases.

## 1. Introduction

The endoscopy is an essential medical examination with various applications in modern clinics [1]. These applications include its role as a biopsy tool, an imaging tool for different body cavities, and a treatment tool during minimally invasive procedures. Rigid endoscopes are among the most important endoscopes for medical diagnosis since the advent of endoscopy [2]. Despite the rapid advances in optical fiber and complementary metal oxide semiconductor (CMOS) technologies that have driven the development of flexible endoscopes, rigid endoscopes still offer more precise use control, easier biopsies, and broader accessibility to certain regions [3]. Furthermore, the quality of images obtained via rigid endoscope lens relays still surpasses the fiber-relayed or CMOS-generated images obtained via flexible endoscopes [4,5]. Therefore, rigid endoscopy is still preferred over flexible endoscopy whenever possible [6,7]. One such field where rigid endoscopy is preferred is laparoscopic surgery [8], in which a single endoscope inserted through a trocar canal provides the necessary information. However, when combined with image processing, the user can obtain more information about the desired area, which can assist in surgery [1].

Various imaging modalities have been combined with endoscopy to identify diseased areas by visualizing certain body parameters [9,10]. In particular, blood flow and oxygen saturation of localized blood flow are essential for tissue viability [11]. Visualization of blood flow and oxygen saturation enables users to identify ischemic tissues. Considering that prolonged ischemia can lead to severe ischemia–reperfusion injury or even tissue necrosis [12,13], preventing and visualizing ischemic tissue is essential for preventing complications.

Laser speckle contrast imaging (LSCI) and hemodynamic response imaging are noninvasive imaging techniques commonly used to evaluate ischemia in real time. LSCI, which provides real-time visualization of blood flow within biological tissues, is based on the analysis of the speckle patterns produced by the interaction between coherent laser light with the tissue, the speckle patterns of which reflect the movement of red blood cells (RBCs) [14]. Hemodynamic response imaging measures blood oxygenation and volume in tissues by detecting the ratio of reflected light at the absorption spectrum of oxygenated and deoxygenated hemoglobin molecules (HbO and HbR, respectively). While LSCI allows for the assessment of microcirculation and has been particularly useful in detecting blood flow changes related to pathologies such as ischemia [15,16,17], hemodynamic response imaging is useful for evaluating tissue perfusion and oxygen delivery and detecting regions of reduced blood flow. Together, LSCI and hemodynamic response imaging provide complementary information on tissue perfusion and oxygenation, which are critical for detecting ischemic conditions, such as following a surgical transplant procedure [18].

Given their inexpensive nature and simple algorithms, there have been many attempts to incorporate LSCI and hemodynamic techniques into portable point-of-care (POC) devices [19,20,21,22]. Similarly, smartphones have emerged as a valuable resource for POC device adaptation.

The applications of smartphones are being extended into medical POC devices. With their powerful processors and camera performance, most smartphone-based POC devices are heavily centered around camera function [23,24,25]. As a result, developers have attempted to create smartphone-based POC endoscope devices. Previous such devices often substituted the camera components of an endoscope with a smartphone rather than maximizing the utility of the smartphone as a powerful image-processing device let alone incorporating other functions such as high-speed video capture [26,27]. Although there have been attempts to utilize the high-speed capture function of newer smartphones [28] to visualize vocal cord movement, image processing was typically performed on a separate device, such as a computer. Furthermore, there was no way to view the processed image in real time, making it less useful in tasks requiring real-time visualization such as surgery. Image processing methods such as LSCI and hemodynamics are used to measure blood flow and oxygen saturation in the medical field in real time.

Here, we built an LSCI and hemodynamic response imaging endoscope device based on the high-speed camera function of a modern smartphone. We also developed an accompanying smartphone application to control the device and complement its functionality. The device’s functionality was evaluated through in vitro polydimethylsiloxane (PDMS) phantom imaging experiments and in vivo rat intestinal vein clamping ischemia experiments. The incorporation of the complementary imaging techniques of LSCI and hemodynamic imaging into a single device can potentially improve surgical outcomes by enabling the early detection of ischemic events during transplant surgeries.

## 2. Materials and Methods

### 2.1. Smartphone-Based Rigid Endoscope System Combining LSCI and Hemodynamic Response Imaging

**Figure 1:**
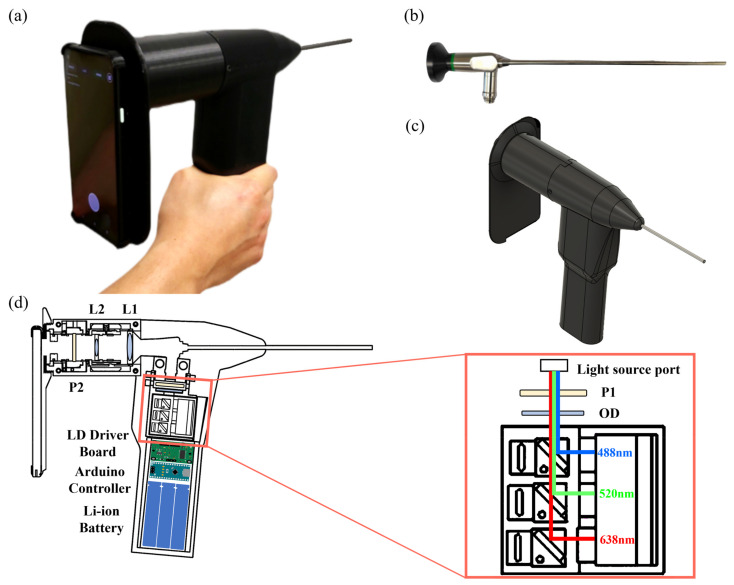
Smartphone-based rigid hemodynamic and laser speckle contrast imaging device. (**a**) Constructed smartphone rigid endoscope. (**b**) Rigid endoscope for the detachable endoscope part of the smartphone-based rigid endoscope device. (**c**) Three-dimensional (3D) model of the device. (**d**) Composition of optical and electrical system built in the smartphone-based rigid endoscope device.

We developed a multimodal smartphone-based rigid endoscope device. As shown in Figure 1, the device is handheld and built around a rigid endoscope using three-dimensionally (3D) printed parts. Figure 1c presents a 3D design of the system consisting of a smartphone rigid endoscope adapter with optical elements and electronics with a laser light source. The interior of the system (Figure 1d) illustrates the relative positioning of the incorporated RGB laser source module, the rigid endoscope, and optical components.

The RGB laser source module (Opt Laser, 300 mW Micro RGB Laser Module) contains a 638 nm 150 mW red laser diode, 520 nm 70 mW green laser diode, 488 nm 45 mW blue laser diode, and laser diode module driver for operating the lasers. The RGB laser source module is controlled by an Arduino microcontroller board (Arduino, Arduino Nano, 16 MHz, 5 V) within the device and powered by lithium-ion rechargeable batteries. Between the optical source port of the rigid endoscope and laser output port of the RGB laser module, two optical elements are utilized to enhance the imaging quality. The optical diffuser OD (Thorlabs, ED1-C20-MD) is used to ensure uniform laser illumination, while an optical polarizer P1 (Thorlabs, LPVIS100-MP2) is used to eliminate specular reflection. The required lenses, diffusers, and polarizers were acquired from Thorlabs (Newton, NJ, USA).

The optical components in addition to the rigid endoscope and smartphone camera consist of two achromatic lenses (L1, L2) and a second optical polarizer P2 (Thorlabs, LPVIS100-MP2). The combination of L1 (Thorlabs, AC254-050-A-ML, f = 50 mm) and L2 (Thorlabs, C330TMD-A, f = 3.1 mm) serves as relay optics to effectively transmit light from the camera connector image surface of the rigid endoscope to the focusing lens on the smartphone camera. In minimize strong specular reflection on the sample surface, a pair of orthogonal linear polarizers (P1, P2) are positioned in front of the RGB laser module and the smartphone camera, respectively. A commercially available laryngoscope (Hinode Light, China) is adapted for use as the rigid endoscope in our system. The rigid endo-scope features a 0°, 4 mm diameter, 175 mm long endoscope probe, a light port compatible with Wolf and Storz, and a camera connector.

A commercially available Android smartphone (Pixel 4a; Google, CA, USA) was mounted on the endoscope using a 3D-printed smartphone adapter. The final endoscopic video acquired 120 fps at a resolution of 1024 pixels × 720 pixels for postprocessing. Figure 1a shows the implemented system as developed. The finial system has dimensions of 60 mm (width) × 180 mm (height) × 280 mm (length) and weighs less than 2 kg.

The developed multimodal system can acquire three imaging modes using a custom-made smartphone application (described in the following Section 2.2). Essentially, the system can illuminate three different wavelength lasers simultaneously; therefore, the system user can monitor the bright field endoscopy images. Through the Arduino control board, the system can illuminate only the 638 nm red laser on the sample surface, and only image data from this red 638 nm laser provides LSCI information. The 638 nm laser speckle video data contains temporal speckle noise pattern changes caused by the flowing scatters, such as RBCs, which can be converted to flow information [29]. To describe the degree of speckle fluctuations, the speckle contrast *K* is typically defined as the ratio of the standard deviation of the light intensities over a region to the mean value ranging from 0 to 1 proportional to the blood flow.

Among the strategies used to calculate the speckle contrast K, we adopted a temporal laser speckle contrast analysis (TLASCA) method [30,31], which offers the advantage of reducing image noise while preserving acceptable spatiotemporal resolution. To implement the TLASCA algorithm, the 15 captured raw speckle images (1024 × 720 × 15 pixels) were converted to grayscale images. The mean and standard deviation of the intensities at all pixels in each image pixel positions were calculated, and their ratio generated the speckle contrast K. Consequently, at the completion of the sequential image calculation, the output of K values formed a speckle contrast image representing flow velocity. Prior to the LSCI and hemodynamic calculations, raw images were postprocessed in the Android application, while filtering for noise reduction and intensity was normalized, including moving average.

We can also analyze hemodynamic information by using the simultaneously captured RGB video. Most existing multichannel wavelength hemodynamics monitoring systems apply switching mechanisms for the light source or the detection channel to obtain light intensity information for each wavelength band. However, our system calculates hemodynamic information using continuous images without switching light sources or filters. The developed system illuminates only the 488 nm laser and the 638 nm laser during the acquisition of hemodynamic video data. With reference to the camera sensor sensitivity spectrum, we minimized interchannel interference by selecting only the blue 488 nm channel and red 638 nm channel to calculate hemodynamic changes. The image reconstruction of oxy hemoglobin and deoxy hemoglobin concentration change use the diffuse reflectance difference of oxy- and deoxyhemoglobin by wavelength. This calculation model is based on modified Beer–Lambert law, and by using a modified Beer–Lambert law, relative concentration changes of oxy- and deoxyhemoglobin are defined as follows [32]:

$$[\begin{array}{c} \Delta [{\mathrm{HbO}}_{2}\left(t\right)]\\ \Delta \left[\mathrm{HbR}\left(t\right)\right] \end{array}]=[\begin{array}{cc} \xi_{{\mathrm{HbO}}_{2}}{}^{\lambda_{1}} & \xi_{\mathrm{HbR}}{}^{\lambda_{1}}\\ \xi_{{\mathrm{HbO}}_{2}}{}^{\lambda_{2}} & \xi_{\mathrm{HbR}}{}^{\lambda_{2}} \end{array}{]}^{-1}[\begin{array}{c} \frac{\ln\left(I_{\lambda_{1}}\left(t_{0}\right)/I_{\lambda_{1}}\left(t\right)\right)}{{\mathrm{DPFx}}_{\lambda_{1}}}\\ \frac{\ln\left(I_{\lambda_{2}}\left(t_{0}\right)/I_{\lambda_{2}}\left(t\right)\right)}{{\mathrm{DPFx}}_{\lambda_{2}}} \end{array}]$$

where $I_{\lambda }\left(t_{0}\right)$ is the detected light intensity of particular wavelength at baseline, $I_{\lambda }\left(t\right)$ is the detected light intensity of particular wavelength at measured time point, $\xi_{\;}$ is the absorption coefficient of a specific chromophore at a particular wavelength, DPFx is the mean pathlength of light traveled in biological tissue at a particular wavelength.

Additionally, the change in total hemoglobin concentration Δ[HbT] can be calculated as follows:

Δ[HbT] =  Δ[HbO_2_] + Δ[HbR]

Following above equations, we can calculate the relative concentration changes in oxy-, deoxy-, and total hemoglobin and reconstruct the hemodynamic response image.

### 2.2. Installation Development of Smartphone App User Interface for Real-Time Endoscopy and LSCI

**Figure 2:**
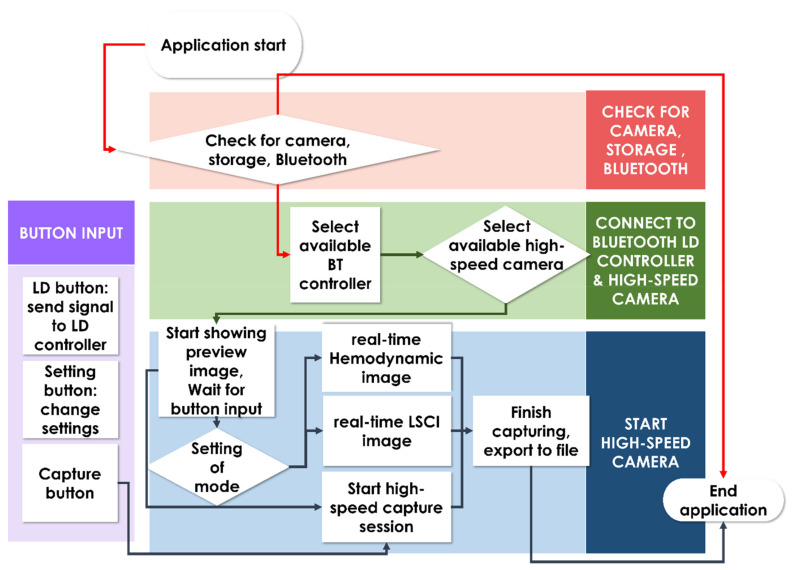
Workflow of the smartphone-based rigid high-speed hemodynamic and LSCI imaging system, illustrated in a simplified flow chart. BT: Bluetooth; LD: laser diode; LSCI: laser speckle contrast imaging.

**Figure 3:**
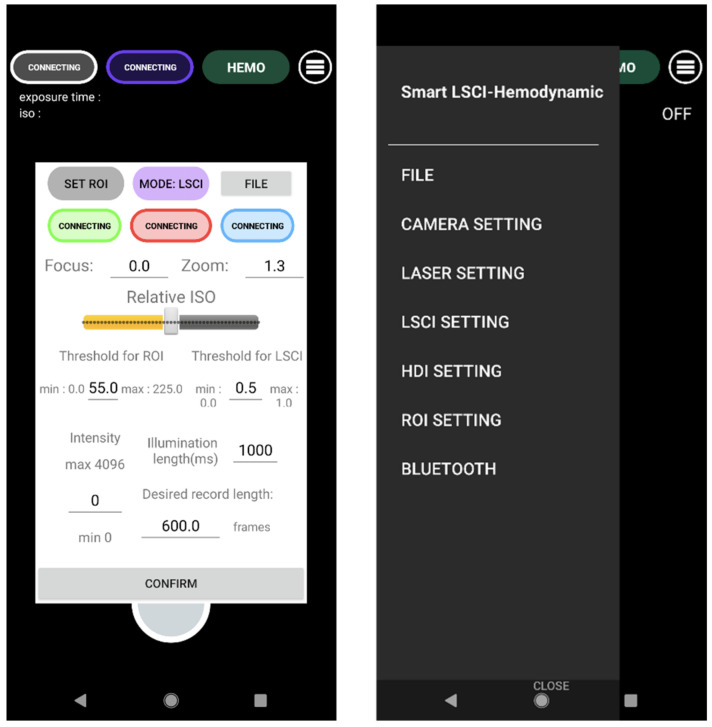
The smartphone application’s user interface allows for control over image capture and laser illumination settings as well as LSCI and hemodynamic image postprocessing. LSCI: laser speckle tracking imaging; ROI: region of interest.

To operate the endoscope device on a smartphone screen, we developed a smartphone application complementing the device. The workflow of the application is briefly described in Figure 2. After checking permissions for the camera, storage, and Bluetooth, the connection to the Bluetooth laser light source control module was established. Upon choosing a camera that was available and suitable for high-speed capture, the preview image is presented, waiting for button input. Figure 3 displays the user interface screen available for main previewing, recording, and real-time image processing of LSCI and hemodynamic response imaging. Developed using Android Studio (Google), the user interface was designed to provide users intuitive control over the device with all controls and ability to adjust settings provided on the screen. The top consists of direct controls over the laser light source and a settings button. The detailed settings menu only appears when the settings button is pressed, thereby reducing visual stress on the user. Pressing the settings menu button toggles detailed settings for the laser light source, Bluetooth connection to the laser light source module, options for LSCI and hemodynamic image processing, and camera settings. For the laser light source, illumination length, intensity, and color options are available. The Bluetooth connection button enables reconnection to the module. The region of interest (ROI) setting enables automatic setting for the ROI. For LSCI and hemodynamic image processing, the threshold of the image and the method of processing can be adjusted. Finally, the camera settings menu enables focus, zoom, record length, and relative intrinsic signal optics value control. When the record button (round button at the bottom) is pressed, recording of high-speed images starts simultaneously; once processed, the LSCI or hemodynamic response image is shown according to the user’s choice. The real-time calculation of the LSCI and hemodynamic response image is facilitated by the OpenCV library implemented in the application.

### 2.3. Tissue-Like Flow Phantom Experiment

To validate the LSCI imaging capabilities of the developed system, we created a tissue-like scattering flow phantom. The flow phantom consists of PDMS mixed with 0.15% (15 g/100 mL) TiO_2_ to simulate an optically scattering medium similar to the in vivo tissue background [33,34]. We used a 1 mm outer diameter metal tube for the flow phantom mold, creating a 1 mm diameter flow channel in the flow phantom. A high precision infusion syringe pump (Fusion 200; Chemyx Inc., Stafford, TX, USA) was used to adjust the flow rate of a 2.5% micro particle solution from 0.023 mL/min (0.5 mm/s flow speed) to 0.094 mL/min (2.0 mm/s flow speed) to simulate the blood flow of a superficial vessel in the biological tissue, and the flow rates were verified using an inverted microscope. A system holder fixed the position of the rigid endoscope probe 5 mm in front of the surface of the flow phantom.

### 2.4. In Vivo Rat Ischemia Experiment

To verify the proper operation of the developed multimodal smartphone-based rigid endoscope system, we applied our system to in vivo rat ischemia experiments. Eight-week-old Sprague–Dawley rats (*n* = 3) were anesthetized by the intravenous injection of a mixture of 0.12% tiletamine and zolazepam and 0.08% xylazine per 200 g of body weight. Each fully anesthetized animal was placed on a temperature-controllable heating pad. We performed a minimally invasive midline laparotomy and exposed the small intestine for ischemia imaging. We tied both sides of imaging target area’s marginal artery and clipped the main artery to mimic the circumstances of ischemia as described elsewhere in the protocol of inducing intestinal ischemia–reperfusion injury in small animals [35]. Also, the developed device was held at a height of 20 mm from the rat’s abdomen to enable imaging of the entire exposed small intestine area. All animal experimental procedures were reviewed and approved by the Institutional Animal Care and Use Committee of Asan Medical Center (protocol no. 2020-12-111) under the Laboratory Animal Law of the Republic of Korea.

## 3. Results

### 3.1. Tissue-Like Flow Phantom Experiment

**Figure 4:**
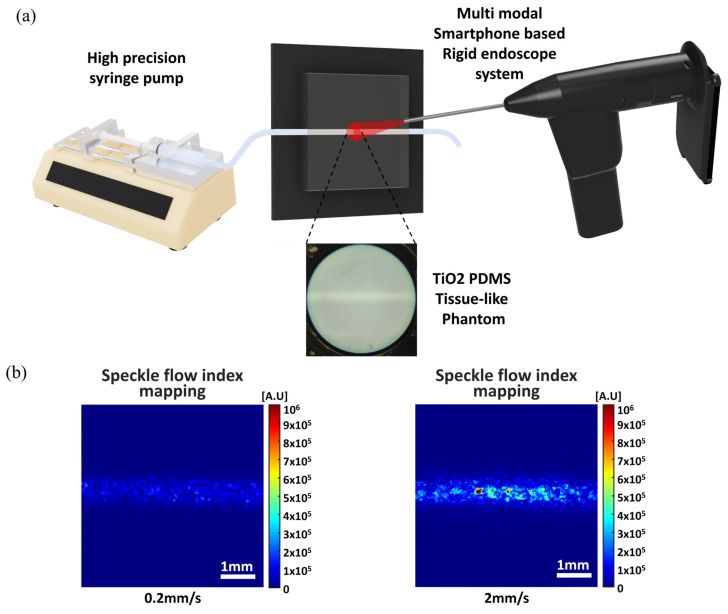
Tissue-like flow phantom test with the smartphone-based multimodal rigid endoscope device. (**a**) The schematic of the flow phantom test. (**b**) Speckle flow index images of the flow phantom at different flow speeds: 0.2 mm/s and 2.0 mm/s. PDMS: polydimethylsiloxane.

Following the design and fabrication of the smartphone-based multimodal rigid endoscope device, we tested the LSCI imaging ability of our device on a tissue-like phantom for blood flow, as illustrated in Figure 4a. Initially, bright field images were acquired using simultaneous RGB laser illumination to allow the focus and illumination intensity to be adjusted in to match the conditions of the tissue-like flow phantom. Utilizing the developed application’s laser diode operation function, the tissue-like flow phantom was then illuminated with a 638 nm red laser for LSCI analysis. Video data with 1024 × 720 pixels resolution were acquired at 120 fps and converted to speckle flow index (SFI) mapping images calculated as 1/(2TK2), where T is the exposure time of the camera and K is the ratio of the speckle to the mean, known as the speckle contrast [36]. In our device’s video acquisition settings, the smartphone camera’s exposure time (T) was 8.33 ms. As shown in Figure 4b, the SFI images of the tissue-like flow phantom at flow speeds of 0.2 mm/s and 2.0 mm/s indicate that higher flow speeds correspond to higher SFI values and lower flow speeds correspond to lower SFI values. These results suggest that our developed system could potentially image perfused structures in biological tissue.

### 3.2. In Vivo Rat Ischemia Experiment

**Figure 5:**
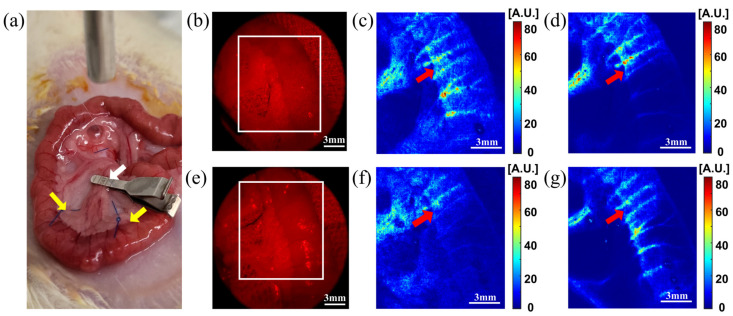
An in vivo rat intestine ischemia model experiment was performed using the LSCI module of the smartphone-based endoscope device. (**a**) Exposed small intestine of an anesthetized rat under the endoscope device (top); the yellow arrows indicate the area of ligated marginal arteries, while the white arrow indicates the clamped main artery of the ischemia-induced region. (**b**) An image of the clamped small intestine illuminated by the 638 nm red laser used for LSCI. (**c**) SFI map before the small intestine is clamped, depicting the ROI marked with a white box shown in (**b**). (**d**) SFI map 10 s after clamping the small intestine in the same ROI. (**e**) An image of the declamped small intestine illuminated by the 638 nm red laser. (**f**) SFI map before declamping of the small intestine from the ROI marked as a white box shown in (**e**). (**g**) SFI map from 10 s after declamping of the small intestine from the same ROI. Red arrows in panels (**c**,**d**,**f**,**g**) indicate the point at which marginal arteries were ligated. LSCI: laser speckle contrast imaging; ROI: region of interest; SFI: speckle flow index.

After verifying the basic laser source control and imaging performance, we conducted in vivo endoscopic blood flow ischemia model imaging of exposed small intestines in normal rats (*n* = 3) to confirm the device’s ability to detect ischemia in a realistic setting (Figure 5). Figure 5a displays the exposed small intestine of an anesthetized rat under the rigid endoscope frontal tip of the developed device. The marginal arteries in the target image area were tied with polypropylene sutures (indicated by yellow arrows). These suture ties helped block the blood flow in the imaging target area. A stainless-steel clamp was used to clamp the main artery of imaging target area (indicated by white arrows). Before acquiring ischemia video data, macroscopic tissue color changes were used to verify ischemia in the imaging area during artery clamping. After the ischemia model’s usefulness was confirmed, video data were acquired from the smartphone-based rigid endoscope device under 638 nm red laser illumination at the moments of clamping and de-clamping (Figure 5b,e). Figure 5c,d present representative rigid endoscopic LSCI analysis SFI mapping results of the clamping situation (mimicking ischemia), while Figure 5f,g show representative rigid endoscopic LSCI analysis SFI mapping results of the declamping situation (mimicking ischemia recovery). The raw video data (6 mm diameter field of view) displays the 638 nm red laser speckle image and suture ties for blocking marginal artery flow. Figure 5c,d show temporal speckle flow indexing map images from an ROI (white boxed area) in Figure 5b, while Figure 5f,g show temporal speckle flow indexing map image from an ROI (white boxed area) in Figure 5e. The red arrows in Figure 5c,d,f,g mark the region of the sutured ligation of the marginal artery. The major artery clamping area was positioned at the bottom of the image’s field of view. Therefore, the area in which blood flow was affected by clamping of the artery was located below the area marked by red arrows in Figure 5c,d,f,g. We conducted a hemodynamic ischemia model experiment using an RGB laser light source in a method identical to the LSCI image acquisition in the ischemia model experiment.

**Figure 6:**
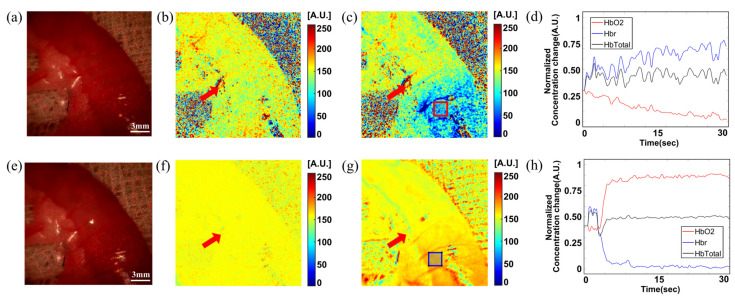
In vivo rat intestine ischemia model hemodynamic imaging using the smartphone-based endoscope device. (**a**) The white light image in LSCI system before clamping of the small intestine. (**b**) Hemodynamic (HbO_2_) concentration change map before clamping of the small intestine. (**c**) HbO_2_ concentration change map after clamping of the small intestine. (**d**) HbO_2_ concentration changes while clamping the small intestine, from the ROI marked with a red box in (**c**). (**e**) The white light image before declamping of the small intestine. (**f**) HbO_2_ concentration change map image before declamping of the ischemic small intestine. (**g**) HbO_2_ concentration change map image after declamping of the small intestine. (**h**) HbO_2_ concentration changes during declamping of the small intestine from the ROI marked with a blue box in (**g**). The red arrows in (**b**,**c**,**f**,**g**) indicate the point at which the marginal arteries are ligated. HbO_2_: hemoglobin oxygenation; ROI: region of interest.

Figure 6 shows the spatial map of relative concentration changes in HbO and HbR in the ischemia and ischemia recovery model as well as of total Hb in specific ROIs. Figure 6a,b present hemodynamic mapping images, while and Figure 6c shows a plot revealing changes in the data as clamping was attempted on the main artery of the small intestine, inducing ischemia. These are contrasted to the changes shown in the recovery model of Figure 6d–f, which portrays hemodynamics after the release of the clamp.

We observed alterations in HbO and HbR concentrations as a result of clamping and declamping, respectively. The red arrows in Figure 6a,b,d,e indicate the point at which blood flow was blocked by ligating the marginal artery of the small intestine using polypropylene sutures. Thus, only the points below the red arrows in each hemodynamic map exhibited changes due to clamping. In the initial mapped image shown in Figure 6a, when clamping was attempted, no significant changes were visible in the target area; however, approximately 30 s later, as shown in the mapping image of Figure 6b, the concentration of HbO in the target area was lower than during the initial clamping. The plot shown in Figure 6c, which represents the average hemoglobin concentration changes within the red square ROI of Figure 6b, shows that the total hemoglobin concentration remains constant from the beginning of clamping, while the concentration of HbO decreases and that of HbR increases. Figure 6d,e display the mapping images of HbO concentration changes after 15 min of clamping and after attempting declamping, respectively, with Figure 6d showing no observable hemodynamic concentration changes from Figure 6c, since it is before declamping. In Figure 6e, the concentration of HbO in the target area increases approximately 30 s after attempting declamping. Figure 6f plots the average hemoglobin concentration changes in the blue square ROI of Figure 6e, revealing a rapid increase in HbO concentration and a decrease in HbR concentration upon declamping. However, no significant changes in the total hemoglobin concentration were visible before versus after declamping. The observed patterns in the hemodynamic concentration change mapping images and plots during clamping and declamping were similar to those expected in the ischemia mimic model, thereby validating the proper functioning of the smartphone hemodynamic response imaging feature using endoscopy and the RGB laser module. The in vivo ischemia experiment demonstrated the functionality of the multimodal smartphone-based endoscope device for detecting ischemia and ischemia recovery in a realistic setting.

## 4. Discussion

This study aimed to combine a rigid endoscope with a smartphone camera and equip it with an RGB laser module to implement both LSCI and hemodynamic response imaging technologies. The rapid advancement of smartphones has led to remarkable performance and camera capabilities, even in non-flagship devices, that have proven sufficient for creating both LSCI and hemodynamic response imaging, as demonstrated here.

LSCI enables visualization of blood vessels and blood flow, making it a viable surgical assistive tool [37,38,39]. Animal studies using LSCI imaging have aimed to identify intestinal blood vessels in laparoscopic open surgery in pigs [40]. Clinically, LSCI has been used in gastrointestinal laparoscopic surgeries to detect ischemic areas related to anastomotic leakage in the colon [41]. In a previous study, smartphone–endoscope LSCI imaging alone was able to provide vascular images of internal organs, sufficiently fulfilling its role as a surgical adjunct for intraoperative assessment [42].

Although LSCI imaging can visualize blood flow to identify ischemic areas during surgery, it is challenging to determine the extent and location of internal organ damage due to ischemia. Consequently, research began on exploring tissue and HbO imaging technology that could indicate the degree and location of surgical organ damage. Hyperspectral imaging (HSI) has been used clinically to provide tissue and hemoglobin oxygenation information [43,44]. Successful HSI applications include visualizing changes in hemoglobin oxygen saturation to identify hemodynamic defects [45], observing tissue oxygenation changes during leg ulcer treatment [46], and aiding in the determination of actual resection areas during colon resection surgery [47]. Studies have also combined LSCI and HSI imaging to reduce the risk of organ damage during surgery [48].

This study aimed to provide additional functional imaging related to tissue damage and area by visualizing changes in oxygen-saturated hemoglobin and deoxyhemoglobin concentrations in tissue using two wavelength bands of RGB lasers. In vivo ischemia animal model experiments showed that LSCI image changes due to vascular occlusion appeared immediately within seconds, and HbO concentrations decreased gradually over 30 s. While LSCI imaging only observed blood flow changes within blood vessels, hemodynamic response imaging showed the effects on all tissues supplied by the occluded blood vessels, as shown in Figure 6a,b. This indicates that the developed device’s hemodynamic concentration change imaging can provide information on the extent and area of tissue damage that LSCI imaging cannot.

The developed multimodal smartphone–endoscope device features a small size and low cost compared to existing commercial products. Commercial LSCI equipment requires a separate laser light source and high-speed camera, while commercial HSI equipment requires a separate camera. These devices are generally expensive, making them inaccessible to low-income developing countries with limited medical infrastructure [49,50,51,52]. However, our device is a viable surgical assistive and diagnostic tool in these settings.

Furthermore, existing POC endoscopic diagnostic devices often provide only bright field images. However, the device we developed not only provides bright field images but also additional functional imaging (LSCI blood flow images and hemodynamic response images), allowing medical professionals to acquire more diagnostic information in the POC environment. As a result, we expect to improve the diagnostic ability and accuracy in the POC environment, where the use of diagnostic equipment is limited.

The limitation of this study is that the hemodynamic response measurement used here is not based on hyperspectral data calculations but obtained from only two visible wavelengths. This means that it cannot provide absolute tissue and hemoglobin oxygenation values like hyperspectral imaging; rather, it can indicate only the change in concentration from the initial tissue and hemoglobin levels in the acquired image. This imaging method can be referred to as intrinsic signal optics imaging, which is typically used to determine neural activity in a specific area by observing changes in cerebral cortex oxygenation concentration [53,54,55]. As a result, the developed device cannot monitor long-term changes in oxygenation levels and is unable to detect oxygenation changes that occurred prior to image acquisition. However, it can still identify damaged areas in situations such as ongoing tissue damage from persistent bleeding and locate unconnected blood vessels or tissue areas during suturing using LSCI and hemodynamic response imaging. Therefore, we expect that it will be sufficiently effective as a diagnostic and surgical assistive tool.

Future work on this device may focus on refining its design and enhancing its imaging capabilities. This could involve improving the optical resolution, increasing the field of view, and exploring additional imaging modalities, such as fluorescence imaging or multispectral imaging, to provide even more comprehensive information about tissue health. We also plan to conduct further preclinical studies to assess the device’s efficacy in different surgical scenarios and compare its performance with existing clinical imaging tools. The development of lightweight machine learning algorithms offers the possibility of integrating artificial intelligence techniques into this smartphone-based multimodal endoscope to provide real-time analysis during surgeries. This would further assist surgeons in their decision-making process and potentially improve surgical outcomes.

The presented smartphone-based multimodal rigid endoscope has shown potential as a valuable tool for real-time monitoring of tissue oxygenation and blood flow during in vivo endoscopic surgery. With continued advancements in the device’s design and capabilities, it may contribute significantly to the future of minimally invasive surgery and ultimately improve patient care. Figure 4a,b show the regions of blood vessels with both free and clamped flows. The spectrum was monitored for changes in the region indicated by the white dotted line in the figure and the signal change over time was recorded from 600 to 870 nm, as shown for the clamped region in Figure 4c.

Over time, the signal amplitude decreased in the spectral region related to hemoglobin oxidization. Monitoring of the observed target area in 15 min increments showed a uniform and narrow standard deviation of the average spectrum, indicating low variation between measurements. In addition, as shown in Figure 4d, following declamping, the time evolution of the 632 nm signal intensity, which is related to heme oxidation saturation, returned to the same level as that of the control blood vessels.

## 5. Conclusions

Here, we successfully developed a novel smartphone-based multimodal rigid endoscope system capable of acquiring both LSCI and hemodynamic response images. The developed portable smartphone–endoscope system can both observe vascular flow in simulated internal organs via LSCI and analyze tissue perfusion due to tissue damage through hemodynamic response imaging. The integration of LSCI and multispectral imaging into a single device promises to provide surgeons with real-time information on tissue health, allowing them to make better-informed decisions during surgery. Furthermore, the device’s compact and low-cost design, which takes advantage of established rigid endoscopes and commodity smartphones, demonstrates the potential to make these imaging modalities more accessible in a wide range of healthcare settings, benefiting patients in terms of improved surgical outcomes and reduced complications.

## Data Availability

Original and raw data files are available from the authors upon reasonable request.

## References

[1] John J.P., Rodriguez H. (2020). Endoscopy, An Issue of Surgical Clinics.

[2] Kuipers E., Haringsma J. (2005). Diagnostic and therapeutic endoscopy. J. Surg. Oncol..

[3] Gustafson L.M., Tami T.A. (2000). Flexible versus rigid esophagoscopy: A practical comparison for otolaryngologists. Curr. Opin. Otolaryngol. Head Neck Surg..

[4] Schneider A., Feussner H. (2017). Diagnostic Procedures, Biomedical Engineering in Gastrointestinal Surgery.

[5] Oi S., Shibata M., Tominaga J., Honda Y., Shinoda M., Takei F., Tsugane R., Matsuzawa K., Sato O. (2000). Efficacy of neuroen-doscopic procedures in minimally invasive preferential management of pineal region tumors: A prospective study. J. Neurosurg..

[6] Moore A.H., England G. (2008). Rigid endoscopy: Urethrocystoscopy and vaginoscopy. BSAVA Manual of Canine and Feline Endoscopy and Endosurgery.

[7] Unfried G., Wieser F., Albrecht A., Kaider A., Nagele F. (2001). Flexible versus rigid endoscopes for outpatient hysteroscopy: A prospective randomized clinical trial. Hum. Reprod..

[8] Li Z., Chiu P.W.-Y. (2018). Robotic Endoscopy. Visc. Med..

[9] Forrester K.R., Stewart C., Leonard C., Tulip J., Bray R.C. (2003). Endoscopic laser imaging of tissue perfusion: New instru-mentation and technique. Lasers Surg. Med..

[10] Bray R.C., Forrester K.R., Reed J., Leonard C., Tulip J. (2006). Endoscopic laser speckle imaging of tissue blood flow: Applications in the human knee. J. Orthop. Res..

[11] Kojima S., Sakamoto T., Nagai Y., Matsui Y., Nambu K., Masamune K. (2019). Laser Speckle Contrast Imaging for Intraoperative Quantitative Assessment of Intestinal Blood Perfusion During Colorectal Surgery: A Prospective Pilot Study. Surg. Innov..

[12] Piper H., Meuter K., Schäfer C. (2003). Cellular mechanisms of ischemia-reperfusion injury. Ann. Thorac. Surg..

[13] Park J.L., Lucchesi B.R. (1999). Mechanisms of myocardial reperfusion injury. Ann. Thorac. Surg..

[14] Senarathna J., Rege A., Li N., Thakor N.V. (2013). Laser Speckle Contrast Imaging: Theory, Instrumentation and Applications. IEEE Rev. Biomed. Eng..

[15] Zhang S., Li Y., Liu S., Ma P., Guo M., Zhou E., Duan L., Fan J., Liao T., Tan Q. (2022). Ischemia and reperfusion injury combined with cisplatin induces immunogenic cell death in lung cancer cells. Cell Death Dis..

[16] Mennes O.A., van Netten J.J., van Baal J.G., Steenbergen W. (2019). Assessment of microcirculation in the diabetic foot with laser speckle contrast imaging. Physiol. Meas..

[17] Dunn A.K. (2011). Laser Speckle Contrast Imaging of Cerebral Blood Flow. Ann. Biomed. Eng..

[18] Katsui S., Inoue Y., Yamamoto Y., Igari K., Kudo T., Uetake H. (2018). In Patients with Severe Peripheral Arterial Disease, Re-vascularization-Induced Improvement in Lower Extremity Ischemia Can Be Detected by Laser Speckle Contrast Imaging of the Fluctuation in Blood Perfusion after Local Heating. Ann. Vasc. Surg..

[19] Chen H., Miao P., Bo B., Li Y., Tong S. A prototype system of portable laser speckle imager based on embedded graphics processing unit platform. Proceedings of the 2019 41st Annual International Conference of the IEEE Engineering in Medicine and Biology Society (EMBC).

[20] Kong P., Xu H., Li R., Huang G., Liu W. (2021). Laser Speckle Contrast Imaging Based on a Mobile Phone Camera. IEEE Access.

[21] Eckle T., Gayat E., Aulagnier J., Matthieu E., Boisson M., Fischler M. (2012). Non-Invasive Measurement of Hemoglobin: As-sessment of Two Different Point-of-Care Technologies. PLoS ONE.

[22] Banerjee A., Bhattacharyya N., Ghosh R., Singh S., Adhikari A., Mondal S., Roy L., Bajaj A., Ghosh N., Bhushan A. (2023). Non-invasive estimation of hemoglobin, bilirubin and oxygen saturation of neonates simultaneously using whole optical spectrum analysis at point of care. Sci. Rep..

[23] Xu X., Akay A., Wei H., Wang S., Pingguan-Murphy B., Erlandsson B.-E., Li X., Lee W., Hu J., Wang L. (2015). Advances in Smartphone-Based Point-of-Care Diagnostics. Proc. IEEE.

[24] Hu J., Cui X., Gong Y., Xu X., Gao B., Wen T., Lu T.J., Xu F. (2016). Portable microfluidic and smartphone-based devices for monitoring of cardiovascular diseases at the point of care. Biotechnol. Adv..

[25] Purohit B., Kumar A., Mahato K., Chandra P. (2020). Smartphone-assisted personalized diagnostic devices and wearable sensors. Curr. Opin. Biomed. Eng..

[26] Quimby A.E., Kohlert S., Caulley L., Bromwich M. (2018). Smartphone adapters for flexible Nasolaryngoscopy: A systematic review. J. Otolaryngol. Head Neck Surg..

[27] Alawsi T., Al-Bawi Z. (2019). A review of smartphone point-of-care adapter design. Eng. Rep..

[28] Kim Y., Oh J., Choi S.-H., Jung A., Lee J.-G., Lee Y.S., Kim J.K. (2021). A Portable Smartphone-Based Laryngoscope System for High-Speed Vocal Cord Imaging of Patients with Throat Disorders: Instrument Validation Study. JMIR mHealth uHealth.

[29] Boas D.A., Dunn A.K. (2010). Laser speckle contrast imaging in biomedical optics. J. Biomed. Opt..

[30] Cheng H., Yan Y., Duong T.Q. (2008). Temporal statistical analysis of laser speckle images and its application to retinal blood-flow imaging. Opt. Express.

[31] Ramirez-San-Juan J.C., Regan C., Coyotl-Ocelotl B., Choi B. (2014). Spatial versus temporal laser speckle contrast analyses in the presence of static optical scatterers. J. Biomed. Opt..

[32] Qin J., Reif R., Zhi Z., Dziennis S., Wang R. (2012). Hemodynamic and morphological vasculature response to a burn moni-tored using a combined dual-wavelength laser speckle and optical microangiography imaging system. Biomed. Opt. Express.

[33] Goldfain A.M., Lemaillet P., Allen D.W., Briggman K.A., Hwang J. (2021). Polydimethylsiloxane tissue-mimicking phantoms with tunable optical properties. J. Biomed. Opt..

[34] Hwang J., Kim H.-J., Lemaillet P., Wabnitz H., Grosenick D., Yang L., Gladytz T., McClatchy D., Allen D., Briggman K. (2017). Polydimethylsiloxane tissue-mimicking phantoms for quantitative optical medical imaging standards. Design and Quality for Biomedical Technologies X.

[35] Tumanov A.V., Jobin C., Koroleva E.P., Perez-Chanona E., Gubernatorova E.O. (2016). Murine Model of Intestinal Ische-mia-reperfusion Injury. J. Vis. Exp..

[36] Choi B., Ramirez-San-Juan J.C., Lotfi J., Nelson J.S. (2006). Linear response range characterization and in vivo application of laser speckle imaging of blood flow dynamics. J. Biomed. Opt..

[37] Miller D.R., Ashour R., Sullender C.T., Dunn A.K. (2022). Continuous blood flow visualization with laser speckle contrast imaging during neurovascular surgery. Neurophotonics.

[38] Wildeboer A., Heeman W., van der Bilt A., Hoff C., Calon J., Boerma E.C., Al-Taher M., Bouvy N. (2022). Laparoscopic Laser Speckle Contrast Imaging Can Visualize Anastomotic Perfusion: A Demonstration in a Porcine Model. Life.

[39] Oh E., Kim W.W., Nam S.-H., Cheon G.W., Ning B., Cha J. (2020). Development of a portable imager for intraoperative real-time localization of parathyroid glands. Advanced Biomedical and Clinical Diagnostic and Surgical Guidance Systems XVIII.

[40] Zheng C., Lau L.W., Cha J. (2018). Dual-display laparoscopic laser speckle contrast imaging for real-time surgical assistance. Biomed. Opt. Express.

[41] Heeman W., Dijkstra K., Hoff C., Koopal S., Pierie J.-P., Bouma H., Boerma E.C. (2019). Application of laser speckle contrast imaging in laparoscopic surgery. Biomed. Opt. Express.

[42] Kim Y., Choi W.J., Oh J., Kim J.K. (2022). Compact Smartphone-Based Laser Speckle Contrast Imaging Endoscope Device for Point-of-Care Blood Flow Monitoring. Biosensors.

[43] Lu G., Fei B. (2014). Medical hyperspectral imaging: A review. J. Biomed. Opt..

[44] Calin M.A., Parasca S.V., Savastru D., Manea D. (2013). Hyperspectral Imaging in the Medical Field: Present and Future. Appl. Spectrosc. Rev..

[45] Sicher C., Rutkowski R., Lutze S., von Podewils S., Wild T., Kretching M., Daeschlein G. (2018). Hyperspectral imaging as a possible tool for visualization of changes in hemoglobin oxygenation in patients with deficient hemodynamics—proof of concept. Biomed. Eng. Biomed. Tech..

[46] Wild T., Becker M., Winter J., Schuhschenk N., Daeschlein G., Siemers F. (2018). Hyperspectral imaging of tissue perfusion and oxygenation in wounds: Assessing the impact of a micro capillary dressing. J. Wound Care.

[47] Jansen-Winkeln B., Holfert N., Köhler H., Moulla Y., Takoh J.P., Rabe S.M., Mehdorn M., Barberio M., Chalopin C., Neumuth T. (2019). Determination of the transection margin during colorectal resection with hyperspectral imaging (HSI). Int. J. Color. Dis..

[48] Lee S., Namgoong J.-M., Kim Y., Cha J., Kim J.K. (2022). Multimodal Imaging of Laser Speckle Contrast Imaging Combined with Mosaic Filter-Based Hyperspectral Imaging for Precise Surgical Guidance. IEEE Trans. Biomed. Eng..

[49] Conductscience Laser Speckle Perfusion Imager. https://conductscience.com/lab/laser-speckle-perfusion-imager/.

[50] Omegawave Laser Speckle Perfusion Imager. http://www.omegawave.co.jp/en/products/oz/price.shtml.

[51] Uthoff R.D., Song B., Maarouf M., Shi V.Y., Liang R. (2020). Point-of-care, multispectral, smartphone-based dermascopes for dermal lesion screening and erythema monitoring. J. Biomed. Opt..

[52] Ding H., Chen C., Zhao H., Yue Y., Han C. (2019). Smartphone based multispectral imager and its potential for point-of-care testing. Analyst.

[53] Hillman E.M.C. (2007). Optical brain imaging in vivo: Techniques and applications from animal to man. J. Biomed. Opt..

[54] Morone K.A., Neimat J.S., Roe A.W., Friedman R.M. (2017). Review of functional and clinical relevance of intrinsic signal optical imaging in human brain mapping. Neurophotonics.

[55] Kim E., Anguluan E., Kim J.G. (2017). Monitoring cerebral hemodynamic change during transcranial ultrasound stimulation using optical intrinsic signal imaging. Sci. Rep..

